# *N*-(3-Butyl-4-oxo-1,3-thia­zolidin-2-yl­idene)benzamide

**DOI:** 10.1107/S1600536810020556

**Published:** 2010-06-26

**Authors:** Hua-Rong Zhao, Hai-Yan Wang, Xiang-Wu Meng

**Affiliations:** a Department of Chemistry, Zhejiang University, Hangzhou 310027, People’s Republic of China

## Abstract

In the title compound, C_14_H_16_N_2_O_2_S, the thia­zolidine ring is planar [maximum atomic deviation = 0.0080 (14) Å] and twisted slightly with respect to the phenyl ring, making a dihedral angle of 4.46 (14)°. The butyl group displays an extended conformation, with a torsion angle of 169.4 (4)°. In the crystal structure, weak inter­molecular C—H⋯O hydrogen bonds link the mol­ecules, forming supra­molecular chains.

## Related literature

For the pharmaceutical applications of thia­zolidinones, see: Amin *et al.* (2008 ▶); Ramla *et al.* (2007 ▶). For the synthesis, see: Peng *et al.* (2004 ▶).
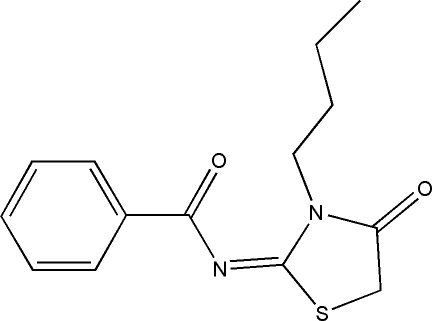


## Experimental

### 

#### Crystal data

C_14_H_16_N_2_O_2_S
*M* *_r_* = 276.35
Monoclinic, 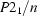

*a* = 5.4690 (1) Å
*b* = 30.5591 (8) Å
*c* = 8.6032 (2) Å
β = 99.895 (3)°
*V* = 1416.44 (6) Å^3^
*Z* = 4
Mo *K*α radiation
μ = 0.23 mm^−1^
*T* = 294 K
0.50 × 0.14 × 0.07 mm

#### Data collection

Oxford Diffraction Nova A diffractometer
Absorption correction: multi-scan (*CrysAlis PRO*; Oxford Diffraction, 2008 ▶) *T*_min_ = 0.895, *T*_max_ = 0.984
7446 measured reflections
2530 independent reflections
1987 reflections with *I* > 2σ(*I*)
*R*_int_ = 0.026

#### Refinement

*R*[*F* ^2^ > 2σ(*F* ^2^)] = 0.044
*wR*(*F* ^2^) = 0.129
*S* = 1.06
2530 reflections
174 parameters
H-atom parameters constrained
Δρ_max_ = 0.20 e Å^−3^
Δρ_min_ = −0.21 e Å^−3^

### 

Data collection: *CrysAlis PRO* (Oxford Diffraction, 2008 ▶); cell refinement: *CrysAlis PRO*; data reduction: *CrysAlis PRO*; program(s) used to solve structure: *SHELXS97* (Sheldrick, 2008 ▶); program(s) used to refine structure: *SHELXL97* (Sheldrick, 2008 ▶); molecular graphics: *ORTEP-3 for Windows* (Farrugia, 1997 ▶) and *OLEX2* (Dolomanov *et al.*, 2009 ▶).; software used to prepare material for publication: *WinGX* (Farrugia, 1999 ▶).

## Supplementary Material

Crystal structure: contains datablocks I, global. DOI: 10.1107/S1600536810020556/sj2789sup1.cif

Structure factors: contains datablocks I. DOI: 10.1107/S1600536810020556/sj2789Isup2.hkl

Additional supplementary materials:  crystallographic information; 3D view; checkCIF report

## Figures and Tables

**Table 1:** Hydrogen-bond geometry (Å, °)

*D*—H⋯*A*	*D*—H	H⋯*A*	*D*⋯*A*	*D*—H⋯*A*
C3—H3⋯O2^i^	0.93	2.39	3.309 (3)	172

Symmetry code: (i) 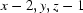.

**Fig. 1.**
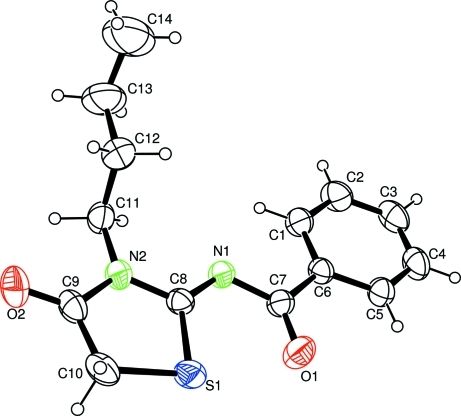
The molecular structure of the title compound with 40% probability displacement ellipsoids.

**Fig. 2.**
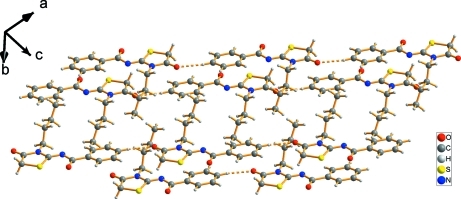
Crystal packing for I viewed down the a axis.

## supplementary crystallographic information

### Comment

Thiazolidinones have wide applications as anticonvulsant (Amin *et al.*, 2008) and anti-neoplastic drugs (Ramla *et al.*, 2007). We report here the structure of a new thiazolidinone derivative, I, Fig. 1.

The thiazolidinyl ring and phenyl ring are almost co-planar with the dihedral angle of 4.46 (14)°. The C11—C12—C13—C14 torsion angle is 169.4 (4)°, showing an extended conformation for the butyl substituent. The N1═C8 bond distance of 1.291 (3) Å  indicates a typical double bond. In the crystal structure, weak intermolecular C—H···O hydrogen bonds, Table 1, link the molecules to form one-dimensional supra-molecular chains, Fig. 2.

### Experimental

The title compound was prepared according to the procedure reported by Peng *et al.* (2004). A 50 ml flask equipped with a dropping funnel was charged with NH_4_SCN (0.152 g, 2 mmol) and [bmim][PF_6_] (2 ml) and was cooled in an ice-water bath. Freshly distilled benzoyl chloride(0.284 g, 2 mmol) was added dropwise and stirred for a further 20 min (disappearance of the starting material was monitored by TLC). n-Butylamine (2 mmol) was then added to the same reaction vessel at room temperature and the mixture was stirred for 20 min more. On completion, ethyl chloroacetate (2.4 mmol) and anhydrous sodium acetate (0.196 g, 2.4 mmol) was added to the flask, and the mixture was heated at 80°C for 2-3 h. After consumption of *N*-benzoyl-*N*'-butylthiourea as indicated by by TLC monitoring, the salts were firstly leached with water (5 ml×2), and the crude product was collected by filtration. Recrystallization from ethanol gave pure product as a yellow crystalline solid.

### Refinement

H atoms were placed in calculated positions with C—H = 0.93 (aromatic), 0.96 (methyl) and 0.97 Å (methine). The torsion angle of methyl group was refined to fit the electron density, with U_iso_(H) = 1.5U_ea_(C). For the other H atoms, U_iso_(H) = 1.2U_eq_(C).

### Crystal data

**Table:**

C_14_H_16_N_2_O_2_S	*F*(000) = 584
*M**_r_* = 276.35	*D*_x_ = 1.296 Mg m^−^^3^
Monoclinic, *P*2_1_/*n*	Mo *K*α radiation, λ = 0.71073 Å
Hall symbol: -P 2yn	Cell parameters from 3174 reflections
*a* = 5.4690 (1) Å	θ = 2.8–25.0°
*b* = 30.5591 (8) Å	µ = 0.23 mm^−^^1^
*c* = 8.6032 (2) Å	*T* = 294 K
β = 99.895 (3)°	Platelet, yellow
*V* = 1416.44 (6) Å^3^	0.50 × 0.14 × 0.07 mm
*Z* = 4	

### Data collection

**Table:**

Oxford Diffraction Nova A diffractometer	2530 independent reflections
Radiation source: fine-focus sealed tube	1987 reflections with *I* > 2σ(*I*)
graphite	*R*_int_ = 0.026
ω scans	θ_max_ = 25.1°, θ_min_ = 1.3°
Absorption correction: multi-scan (*CrysAlis PRO*; Oxford Diffraction, 2008)	*h* = −6→3
*T*_min_ = 0.895, *T*_max_ = 0.984	*k* = −36→36
7446 measured reflections	*l* = −10→10

### Refinement

**Table:**

Refinement on *F*^2^	Secondary atom site location: difference Fourier map
Least-squares matrix: full	Hydrogen site location: inferred from neighbouring sites
*R*[*F*^2^ > 2σ(*F*^2^)] = 0.044	H-atom parameters constrained
*wR*(*F*^2^) = 0.129	*w* = 1/[σ^2^(*F*_o_^2^) + (0.0615*P*)^2^ + 0.3856*P*] where *P* = (*F*_o_^2^ + 2*F*_c_^2^)/3
*S* = 1.06	(Δ/σ)_max_ = 0.001
2530 reflections	Δρ_max_ = 0.20 e Å^−^^3^
174 parameters	Δρ_min_ = −0.21 e Å^−^^3^
0 restraints	Extinction correction: *SHELXL97* (Sheldrick, 2008), Fc^*^=kFc[1+0.001xFc^2^λ^3^/sin(2θ)]^-1/4^
Primary atom site location: structure-invariant direct methods	Extinction coefficient: 0.0113 (18)

### Special details

**Table:**

Geometry. All esds (except the esd in the dihedral angle between two l.s. planes) are estimated using the full covariance matrix. The cell esds are taken into account individually in the estimation of esds in distances, angles and torsion angles; correlations between esds in cell parameters are only used when they are defined by crystal symmetry. An approximate (isotropic) treatment of cell esds is used for estimating esds involving l.s. planes.
Refinement. Refinement of *F*^2^ against ALL reflections. The weighted *R*-factor wR and goodness of fit *S* are based on *F*^2^, conventional *R*-factors *R* are based on *F*, with *F* set to zero for negative *F*^2^. The threshold expression of *F*^2^ > σ(*F*^2^) is used only for calculating *R*-factors(gt) etc. and is not relevant to the choice of reflections for refinement. *R*-factors based on *F*^2^ are statistically about twice as large as those based on *F*, and *R*- factors based on ALL data will be even larger.

### Fractional atomic coordinates and isotropic or equivalent isotropic displacement parameters (Å^2^)

**Table:**

	*x*	*y*	*z*	*U*_iso_*/*U*_eq_	
S1	0.28923 (11)	0.210301 (19)	0.34733 (8)	0.0680 (2)	
N1	0.0399 (3)	0.14912 (6)	0.1466 (2)	0.0568 (5)	
N2	0.4097 (3)	0.12988 (6)	0.3021 (2)	0.0604 (5)	
O1	−0.1307 (3)	0.21815 (6)	0.1475 (2)	0.0846 (6)	
O2	0.7761 (3)	0.11970 (7)	0.4680 (2)	0.0903 (6)	
C1	−0.3441 (4)	0.12483 (8)	−0.0946 (3)	0.0675 (6)	
H1	−0.2120	0.1058	−0.0637	0.081*	
C2	−0.5428 (5)	0.11225 (10)	−0.2086 (3)	0.0834 (8)	
H2	−0.5435	0.0846	−0.2540	0.100*	
C3	−0.7382 (5)	0.14015 (11)	−0.2550 (3)	0.0850 (8)	
H3	−0.8711	0.1313	−0.3310	0.102*	
C4	−0.7377 (4)	0.18069 (11)	−0.1902 (3)	0.0798 (8)	
H4	−0.8697	0.1996	−0.2228	0.096*	
C5	−0.5429 (4)	0.19391 (8)	−0.0765 (3)	0.0674 (6)	
H5	−0.5441	0.2217	−0.0322	0.081*	
C6	−0.3447 (4)	0.16583 (7)	−0.0278 (2)	0.0555 (5)	
C7	−0.1369 (4)	0.18080 (7)	0.0965 (3)	0.0588 (5)	
C8	0.2273 (4)	0.16005 (7)	0.2522 (2)	0.0543 (5)	
C9	0.6056 (4)	0.14333 (9)	0.4145 (3)	0.0669 (6)	
C10	0.5774 (4)	0.19006 (9)	0.4582 (3)	0.0740 (7)	
H10A	0.7154	0.2071	0.4338	0.089*	
H10B	0.5756	0.1925	0.5704	0.089*	
C11	0.4005 (4)	0.08576 (8)	0.2365 (3)	0.0693 (6)	
H11A	0.5683	0.0746	0.2452	0.083*	
H11B	0.3289	0.0870	0.1253	0.083*	
C12	0.2517 (5)	0.05489 (9)	0.3177 (4)	0.0900 (8)	
H12A	0.0871	0.0669	0.3160	0.108*	
H12B	0.3303	0.0516	0.4271	0.108*	
C13	0.2296 (8)	0.01017 (11)	0.2380 (6)	0.1436 (17)	
H13A	0.1831	0.0146	0.1251	0.172*	
H13B	0.3921	−0.0035	0.2564	0.172*	
C14	0.0574 (10)	−0.01942 (15)	0.2872 (8)	0.194 (3)	
H14A	0.0878	−0.0214	0.4002	0.291*	
H14B	0.0767	−0.0478	0.2430	0.291*	
H14C	−0.1084	−0.0091	0.2515	0.291*	

### Atomic displacement parameters (Å^2^)

**Table:**

	*U*^11^	*U*^22^	*U*^33^	*U*^12^	*U*^13^	*U*^23^
S1	0.0674 (4)	0.0599 (4)	0.0716 (4)	−0.0109 (3)	−0.0022 (3)	−0.0083 (3)
N1	0.0544 (10)	0.0541 (10)	0.0564 (10)	−0.0011 (8)	−0.0061 (8)	0.0013 (8)
N2	0.0529 (10)	0.0640 (11)	0.0592 (10)	0.0002 (8)	−0.0045 (8)	0.0004 (9)
O1	0.0866 (12)	0.0620 (10)	0.0931 (13)	0.0110 (8)	−0.0190 (10)	−0.0116 (9)
O2	0.0606 (10)	0.1150 (15)	0.0852 (12)	0.0086 (10)	−0.0155 (9)	0.0012 (11)
C1	0.0633 (13)	0.0679 (14)	0.0656 (14)	−0.0014 (11)	−0.0054 (11)	−0.0005 (11)
C2	0.0786 (17)	0.0852 (18)	0.0781 (17)	−0.0140 (14)	−0.0104 (13)	−0.0076 (14)
C3	0.0620 (15)	0.115 (2)	0.0689 (16)	−0.0187 (15)	−0.0132 (12)	0.0112 (16)
C4	0.0534 (13)	0.102 (2)	0.0792 (17)	0.0026 (13)	−0.0040 (12)	0.0219 (16)
C5	0.0577 (13)	0.0705 (14)	0.0714 (14)	0.0035 (11)	0.0035 (11)	0.0101 (12)
C6	0.0495 (11)	0.0596 (12)	0.0548 (11)	−0.0026 (9)	0.0021 (9)	0.0075 (10)
C7	0.0584 (12)	0.0539 (12)	0.0606 (12)	0.0001 (10)	0.0001 (10)	0.0009 (10)
C8	0.0534 (11)	0.0540 (12)	0.0531 (11)	−0.0059 (9)	0.0027 (9)	0.0013 (9)
C9	0.0506 (12)	0.0865 (17)	0.0590 (13)	−0.0085 (11)	−0.0035 (10)	0.0037 (12)
C10	0.0615 (14)	0.0884 (18)	0.0669 (14)	−0.0221 (12)	−0.0039 (11)	−0.0040 (13)
C11	0.0608 (13)	0.0674 (14)	0.0751 (15)	0.0091 (11)	−0.0013 (11)	−0.0030 (12)
C12	0.0860 (19)	0.0689 (16)	0.114 (2)	0.0021 (14)	0.0156 (17)	−0.0020 (16)
C13	0.144 (3)	0.0650 (19)	0.234 (5)	−0.012 (2)	0.068 (3)	−0.017 (3)
C14	0.179 (5)	0.108 (3)	0.313 (8)	−0.038 (3)	0.093 (5)	−0.040 (4)

### Geometric parameters (Å, °)

**Table:**

S1—C8	1.746 (2)	C5—C6	1.390 (3)
S1—C10	1.805 (2)	C5—H5	0.9300
N1—C8	1.291 (3)	C6—C7	1.492 (3)
N1—C7	1.384 (3)	C9—C10	1.491 (4)
N2—C8	1.372 (3)	C10—H10A	0.9700
N2—C9	1.377 (3)	C10—H10B	0.9700
N2—C11	1.459 (3)	C11—C12	1.495 (4)
O1—C7	1.221 (3)	C11—H11A	0.9700
O2—C9	1.206 (3)	C11—H11B	0.9700
C1—C6	1.379 (3)	C12—C13	1.525 (5)
C1—C2	1.387 (3)	C12—H12A	0.9700
C1—H1	0.9300	C12—H12B	0.9700
C2—C3	1.372 (4)	C13—C14	1.421 (5)
C2—H2	0.9300	C13—H13A	0.9700
C3—C4	1.359 (4)	C13—H13B	0.9700
C3—H3	0.9300	C14—H14A	0.9600
C4—C5	1.377 (3)	C14—H14B	0.9600
C4—H4	0.9300	C14—H14C	0.9600
			
C8—S1—C10	91.59 (11)	N2—C9—C10	111.2 (2)
C8—N1—C7	117.76 (19)	C9—C10—S1	108.30 (15)
C8—N2—C9	117.08 (19)	C9—C10—H10A	110.0
C8—N2—C11	121.66 (17)	S1—C10—H10A	110.0
C9—N2—C11	121.24 (19)	C9—C10—H10B	110.0
C6—C1—C2	119.3 (2)	S1—C10—H10B	110.0
C6—C1—H1	120.4	H10A—C10—H10B	108.4
C2—C1—H1	120.4	N2—C11—C12	112.8 (2)
C3—C2—C1	120.7 (3)	N2—C11—H11A	109.0
C3—C2—H2	119.7	C12—C11—H11A	109.0
C1—C2—H2	119.7	N2—C11—H11B	109.0
C4—C3—C2	120.0 (2)	C12—C11—H11B	109.0
C4—C3—H3	120.0	H11A—C11—H11B	107.8
C2—C3—H3	120.0	C11—C12—C13	111.3 (3)
C3—C4—C5	120.4 (2)	C11—C12—H12A	109.4
C3—C4—H4	119.8	C13—C12—H12A	109.4
C5—C4—H4	119.8	C11—C12—H12B	109.4
C4—C5—C6	120.1 (2)	C13—C12—H12B	109.4
C4—C5—H5	120.0	H12A—C12—H12B	108.0
C6—C5—H5	120.0	C14—C13—C12	116.2 (4)
C1—C6—C5	119.5 (2)	C14—C13—H13A	108.2
C1—C6—C7	121.44 (19)	C12—C13—H13A	108.2
C5—C6—C7	119.0 (2)	C14—C13—H13B	108.2
O1—C7—N1	124.6 (2)	C12—C13—H13B	108.2
O1—C7—C6	121.0 (2)	H13A—C13—H13B	107.4
N1—C7—C6	114.46 (19)	C13—C14—H14A	109.5
N1—C8—N2	119.52 (19)	C13—C14—H14B	109.5
N1—C8—S1	128.62 (17)	H14A—C14—H14B	109.5
N2—C8—S1	111.86 (14)	C13—C14—H14C	109.5
O2—C9—N2	123.1 (2)	H14A—C14—H14C	109.5
O2—C9—C10	125.7 (2)	H14B—C14—H14C	109.5
			
C6—C1—C2—C3	0.1 (4)	C11—N2—C8—N1	1.3 (3)
C1—C2—C3—C4	0.5 (4)	C9—N2—C8—S1	−0.2 (3)
C2—C3—C4—C5	−0.7 (4)	C11—N2—C8—S1	−178.67 (17)
C3—C4—C5—C6	0.3 (4)	C10—S1—C8—N1	−179.2 (2)
C2—C1—C6—C5	−0.5 (4)	C10—S1—C8—N2	0.77 (18)
C2—C1—C6—C7	179.3 (2)	C8—N2—C9—O2	179.6 (2)
C4—C5—C6—C1	0.3 (4)	C11—N2—C9—O2	−1.9 (4)
C4—C5—C6—C7	−179.5 (2)	C8—N2—C9—C10	−0.7 (3)
C8—N1—C7—O1	−1.5 (4)	C11—N2—C9—C10	177.8 (2)
C8—N1—C7—C6	178.87 (18)	O2—C9—C10—S1	−179.1 (2)
C1—C6—C7—O1	175.0 (2)	N2—C9—C10—S1	1.2 (3)
C5—C6—C7—O1	−5.1 (3)	C8—S1—C10—C9	−1.12 (19)
C1—C6—C7—N1	−5.3 (3)	C8—N2—C11—C12	−85.8 (3)
C5—C6—C7—N1	174.51 (19)	C9—N2—C11—C12	95.8 (3)
C7—N1—C8—N2	−178.42 (19)	N2—C11—C12—C13	175.6 (3)
C7—N1—C8—S1	1.5 (3)	C11—C12—C13—C14	−169.4 (4)
C9—N2—C8—N1	179.8 (2)		

### Hydrogen-bond geometry (Å, °)

**Table:**

*D*—H···*A*	*D*—H	H···*A*	*D*···*A*	*D*—H···*A*
C3—H3···O2^i^	0.93	2.39	3.309 (3)	172

Symmetry codes: (i) *x*−2, *y*, *z*−1.

## References

[bb1] Amin, K. M., Rahman, D. E. A. & Al-Eryani, Y. (2008). *Bioorg. Med. Chem.* **16**, 5377-5388. 10.1016/j.bmc.2008.04.021 18467106

[bb2] Dolomanov, O. V., Bourhis, L. J., Gildea, R. J., Howard, J. A. K. & Puschmann, H. (2009). *J. Appl. Cryst.* **42**, 339–341.

[bb3] Farrugia, L. J. (1997). *J. Appl. Cryst.* **30**, 565.

[bb4] Farrugia, L. J. (1999). *J. Appl. Cryst.* **32**, 837–838.

[bb5] Oxford Diffraction (2008). *CrysAlis PRO* Oxford Diffraction Ltd, Yarnton, Oxfordshire, England.

[bb6] Peng, Y.-Q., Song, G.-H. & Huang, F.-F. (2004). *J. Chem. Res.* **10**, 676–678.

[bb7] Ramla, M. M., Omarm, M. A., Tokuda, H. & El-Diwani, H. I. (2007). *Bioorg. Med. Chem.* **15**, 6489–6496. 10.1016/j.bmc.2007.04.010 17643992

[bb8] Sheldrick, G. M. (2008). *Acta Cryst.* A**64**, 112–122. 10.1107/S0108767307043930 18156677

